# Ischemia-Reperfusion Injury in Marginal Liver Grafts and the Role of Hypothermic Machine Perfusion: Molecular Mechanisms and Clinical Implications

**DOI:** 10.3390/jcm9030846

**Published:** 2020-03-20

**Authors:** Zoltan Czigany, Isabella Lurje, Moritz Schmelzle, Wenzel Schöning, Robert Öllinger, Nathanael Raschzok, Igor M. Sauer, Frank Tacke, Pavel Strnad, Christian Trautwein, Ulf Peter Neumann, Jiri Fronek, Arianeb Mehrabi, Johann Pratschke, Andrea Schlegel, Georg Lurje

**Affiliations:** 1 Department of Surgery and Transplantation, University Hospital RWTH Aachen, 52074 Aachen, Germany; zczigany@ukaachen.de (Z.C.); uneumann@ukaachen.de (U.P.N.); 2 Department of Surgery, Campus Charité Mitte | Campus Virchow-Klinikum—Charité-Universitätsmedizin Berlin, 13353 Berlin, Germany; isabella@lurje.net (I.L.); moritz.schmelzle@charite.de (M.S.); wenzel.schoening@charite.de (W.S.); robert.oellinger@charite.de (R.Ö.); nathanael.raschzok@charite.de (N.R.); igor.sauer@charite.de (I.M.S.); johann.pratschke@charite.de (J.P.); 3 Department of Hepatology and Gastroenterology, Campus Charité Mitte | Campus Virchow-Klinikum—Charité-Universitätsmedizin Berlin, 13353 Berlin, Germany; frank.tacke@charite.de; 4 Department of Gastroenterology, Metabolic Disorders and Intensive Care, University Hospital RWTH Aachen, 52074 Aachen, Germany; pstrnad@ukaachen.de (P.S.); ctrautwein@ukaachen.de (C.T.); 5 Department of Transplant Surgery, Institute for Clinical and Experimental Medicine, 140 21 Prague, Czech Republic; jifr@ikem.cz; 6 Department of General, Visceral and Transplantation Surgery, University of Heidelberg, 69120 Heidelberg, Germany; arianeb.mehrabi@med.uni-heidelberg.de; 7 The Liver Unit, Queen Elizabeth Hospital Birmingham, Birmingham B15 2TH, UK; andrea.schlegel@outlook.com

**Keywords:** ischemia-reperfusion injury, extended criteria donation, machine perfusion, HOPE, hypothermic machine perfusion

## Abstract

Ischemia-reperfusion injury (IRI) constitutes a significant source of morbidity and mortality after orthotopic liver transplantation (OLT). The allograft is metabolically impaired during warm and cold ischemia and is further damaged by a paradox reperfusion injury after revascularization and reoxygenation. Short-term and long-term complications including post-reperfusion syndrome, delayed graft function, and immune activation have been associated with IRI. Due to the current critical organ shortage, extended criteria grafts are increasingly considered for transplantation, however, with an elevated risk to develop significant features of IRI. In recent years, ex vivo machine perfusion (MP) of the donor liver has witnessed significant advancements. Here, we describe the concept of hypothermic (oxygenated) machine perfusion (HMP/HOPE) approaches and highlight which allografts may benefit from this technology. This review also summarizes clinical applications and the main aspects of ongoing randomized controlled trials on hypothermic perfusion. The mechanistic aspects of IRI and hypothermic MP—which include tissue energy replenishment, optimization of mitochondrial function, and the reduction of oxidative and inflammatory damage following reperfusion—will be comprehensively discussed within the context of current preclinical and clinical evidence. Finally, we highlight novel trends and future perspectives in the field of hypothermic MP in the context of recent findings of basic and translational research.

## 1. Introduction

Liver ischemia-reperfusion injury (IRI) poses a universal risk of adverse clinical outcomes in extended liver resections and orthotopic liver transplantation (OLT). The development of hepatic IRI after OLT has been associated with perioperative complications, including post-reperfusion syndrome, delayed graft, and primary non-function as well as acute rejection [1,2].

The overall success of liver transplantation has resulted in an increasing spectrum of indications [3,4]. Based on the critical organ shortage, an increasing number of extended criteria donor (ECD) allografts are utilized today, which were previously considered unsuitable for transplantation [5,6]. Frequently employed ECD characteristics include advanced donor age, prolonged cold storage of >12 h, macrosteatosis of >30%, mixed steatosis of >60%, organ dysfunction at procurement, and donation after circulatory death (DCD) [7]. Despite the positive impact on waiting list times, the utilization of ECD livers has also been linked to inferior posttransplant outcomes due to their increased susceptibility to IRI and a subsequently reduced ability for functional recovery [8,9,10]. 

The key steps of IRI occur at several timepoints during solid organ transplantation. In an oxygen-depleted graft, reperfusion triggers an inflammatory cascade followed by a subsequent acute and chronic downstream graft injury [11,12]. Various cellular and subcellular compartments are involved which results, for example, in hepatocyte necrosis and endothelial cell apoptosis [13]. 

Static cold storage (SCS) sufficiently sustains most allografts during organ transport and has remained the standard of organ preservation for more than three decades [14]. In ECD allografts, however, SCS imposes an increased susceptibility to IRI and allograft related complications; as such, dynamic preservation techniques including machine perfusion (MP) have evolved as important tools to preserve and recondition such livers [6]. While normothermic machine perfusion (NMP) aims to closely mimic in vivo conditions with the use of oxygenated blood-based perfusate or artificial oxygen carriers, hypothermic machine perfusion (HMP) relies on the use of artificial, cooled perfusion solutions with oxygenation. 

This review summarizes the underlying mechanisms of IRI and the impact of HMP in the transplantation of ECD livers. Additionally, current clinical applications and future perspectives of hypothermic liver perfusion are detailed. 

## 2. Ischemia-Reperfusion Injury and Marginal Allografts

### 2.1. The Mechanism of Ischemia-Reperfusion Injury

Ischemia-reperfusion injury is characterized by three chronological events: The phase of ischemia, followed by the reperfusion, and late or latent injury [15]. In OLT, injury accumulates during the ischemic phase and significantly depends on the type of organ donation (donation after brain (DBD) vs. circulatory death (DCD)) [16]. The next event is the organ reperfusion, which immediately occurs when an organ is reoxygenated under normothermic conditions, either following implantation or ex situ on a normothermic perfusion device. When oxygen is introduced to hypoxic and energy depleted tissue, reactive oxygen species (ROS) are immediately released from the mitochondrial respiratory chain. This key event triggers further downstream inflammation and links the acute phase of IRI to the third component of injury: the activation of resident and recruited immune cells. During this recovery phase of IRI, the newly transplanted solid organ is challenged further through interaction with immunosuppressive drugs, infections, and potential surgical complications [17]. 

Standard cold storage (SCS) relies on hypothermia to decelerate cell metabolism and reduce the oxygen demand of the allograft. However, the cellular metabolism does not cease completely during SCS, and continues as anaerobic metabolism at a low rate, with subsequent depletion of adenosine triphosphate (ATP) stores and the generation of catabolic ATP products [18]. Simultaneously, the respiratory chain, redox active enzymes, and electron carrier pools including NADH and CoenzymeQ_10_ (CoQ) are reduced [19]. It has been suggested that ischemia increases intracellular cAMP levels, which in turn stimulate glycolysis, with subsequent accumulation of hexose-6-phosphates and lactate [20]. Significant cellular ATP depletion results in ionic deregulation due to failure of membranous sodium-potassium (Na+-K+) ATPases, calcium accumulation, and an acidic pH [21,22].

The reintroduction of oxygen to the ischemic tissue following allograft revascularization leads to massive mitochondrial reactive oxygen species (ROS) production and release [19]. Studies on ROS release from the mitochondrial respiratory chain have delineated Complex I as a major source of ROS formation [23,24]. The mitochondrial injury causes further energetic depletion, an impaired mitochondrial calcium buffering capacity, and triggers cell death by ROS released through the mitochondrial permeability transition pore (MPTP) with subsequent danger-associated molecular patterns (DAMPs) released from the nucleus [25]. DAMPs in turn activate Kupffer cells and other non-parenchymal cells through reaction with Toll-like receptors (TLRs) [26]. This provokes a sustained inflammation dominated by innate immune cells, particularly Kupffer cells, which results in the recruitment and activation of additional neutrophil leukocytes and monocytes [27,28,29,30]. Essential microstructures of the liver including the endothelial glycocalyx are highly susceptible to oxidative stress and deteriorate quickly in an ischemic setting [31], leading to further leukocyte and platelet adherence, aggregation, and activation [32]. Furthermore, the discrepancy between vasoconstrictive mediator accumulation and suppression of nitric oxide (NO) levels impairs the microcirculation and perfusion of the liver [33,34], and further aggravates the allograft injury. 

The clinical effects of IRI can manifest as a broad spectrum of short-term and long-term complications, resulting in an overall inferior patient survival [5,6]. IRI-induced complications include intraoperative post-implantation hemodynamic instability (post-reperfusion syndrome), delayed graft function (DGF), and primary nonfunction (PNF), with the latter being the most serious posttransplant complication requiring retransplantation and frequently leading to patient death [6]. Severe IRI furthermore contributes to acute cellular rejection (ACR) according to Lund’s “injury hypothesis”, where the innate inflammatory immune response during IRI provokes a response of the adaptive immune system [1].

### 2.2. The Heterogeneity of ECD Grafts

The term ECD comprises a heterogenous group of different graft types with distinct functional impairments and an increased susceptibility to IRI [6,35]. Based on the growing obesity epidemic, steatotic grafts have emerged as an essential part of the ECD donor pool [36]. Fatty livers—particularly with high percentages of macrosteatosis—are not only prone to develop severe IRI due to their metabolic ATP depletion, but also have an impaired microcirculation [37], and are more susceptible to oxidative stress [38]. Such features create an intrinsically pro-inflammatory microenvironment [39,40]. Both vasoconstriction and excessive ROS formation exacerbate the microcirculatory dysfunction and consecutively activate the innate immune system and lead to massive Kupffer cell activation with neutrophil infiltration and subsequent ACR or chronic graft dysfunction [36,41]. Due to this susceptibility to IRI, steatotic grafts are thought to especially benefit from MP, with the optimal application of MP for steatotic livers currently being evaluated [5,36,42]. Hypothermic perfusion has been shown to improve the metabolic status and recharge energy stores, leading to improved graft survival compared to unperfused macrosteatotic human and rodent livers [42]. However, further clinical evidence is needed in this field. At normothermic temperatures, reoxygenation of the fatty allograft leads to significant IRI. As such, the role of normothermic organ “reconditioning” and defatting protocols remains controversial [36].

In DCD livers, the additional period of in-situ donor warm ischemia time exposes the recipient to a higher risk of developing ischemic-type biliary lesions (ITBLs). The underlying mechanism is multifactorial and includes an impairment of the microcirculation with subsequent ischemic injury of the peribiliary arterial plexus and the formation of non-anastomotic biliary strictures (NAS) [43]. This type of injury remains a critical determinant of the long-term outcome of OLT using DCD allografts [44]. Energy depletion further contributes to the development of ITBLs though the impaired function of ATP dependent hepatocyte bile acid transporters [45]. The initial bile production following reperfusion appears to have a toxic effect, conferring further injury to the cholangiocytes and the entire biliary tree [46]. 

In the context of the demographic development in the Western world, elderly donors have likewise become an important part of the donor pool. Various reports demonstrate that transplantation of elderly donor allografts (>80 years) is safe and not associated with inferior overall outcomes per se [47,48]. Septuagenarian and octogenarian donor livers are successfully utilized with acceptable results [49,50]. However, older livers have a reduced functional reserve and regeneration capacity and are therefore more vulnerable to IRI injury; they show a higher incidence of biliary and arterial complications as well as inferior initial graft function [49]. This graft category was shown to benefit from ex vivo MP to mitigate the impact of additional risk factors such as prolonged periods of cold ischemia [49]. 

## 3. Machine Liver Perfusion Technology

### 3.1. History and Clinical Application

Machine perfusion with oxygenated blood was already implemented in the first series of 11 successful human OLTs in the 1960s [51]. However, the logistical simplicity and reliable performance of SCS led to its quick adoption as the standard solid organ preservation technique in clinical practice. The increased utilization of high-risk organs has unveiled the limitations of SCS, furthering the debate on the impact of different MP techniques. Today, perfusion conditions vary broadly, especially in preclinical research. Parameters under discussion include different temperatures, perfusate composition, the application of dynamic perfusion (continuous or pulsatile), the timing and duration of the perfusion, starting either at the donor site or applied only end-ischemic in the recipient center. Three main principles have been translated into clinical practice today: hypothermic MP (HMP), hypothermic oxygenated perfusion (HOPE) with active oxygenation, and normothermic MP (NMP). The latter differs significantly from the other two hypothermic approaches because the allograft is perfused with oxygenated blood or acellular oxygen carriers at physiological temperatures with the aim to reduce the ischemic graft injury by minimizing the duration of cold preservation and perfectly mimicking physiological conditions [52]. Normothermic perfusion is most effective when applied during the entire period of organ preservation [53], while end-ischemic applications in marginal DBD and DCD livers failed to protect from the development of ITBLs and subsequent graft loss [54]. An additional challenge is the graft transportation on an NMP device because a perfusion standstill will inevitably result in graft damage by warm ischemia. 

A recently completed randomized controlled trial (RCT) by Nasralla et al. proved the feasibility of NMP for OLT and demonstrated a significant reduction in peak aspartate aminotransferase (AST) and subsequent early allograft dysfunction (EAD), however without a significant difference in graft and patient survival [55]. Notably, the cold storage control group achieved a 96% one-year survival rate [55,56]. A development in the NMP technique that allowed a 7-day preservation of human livers with sustained metabolic function and intact liver structure was recently reported by Eshmuminov et al. [57]. Based on the sustained full hepatic metabolism during NMP, several groups are currently exploring the possibility of normothermic viability testing [58]. The cellular mechanisms of organ protection by NMP do not center around IRI mitigation and reconditioning, but IRI prevention, and are altogether different from cold perfusion techniques.

### 3.2. Hypothermic Machine Perfusion

During HMP, perfusion solution circulates through the liver at 4–11 °C, delivering metabolic substrates and washing out metabolic waste products [56]. The perfusate is not actively oxygenated, and therefore has a pO_2_ of approximately 20 kPa [59]. Based on evidence from a large, multicentric RCT that demonstrated superior kidney function, HMP from retrieval to implantation (for example with the LifePort^®^ device) has become routine clinical practice in some European countries in the transplantation of extended criteria kidneys [60]. HMP without additional oxygenation was first described by James Guarrera, who published the first clinical series already in 2010, proving mainly feasibility and safety. Although graft function and survival rates were comparable between HMP-treated livers and unperfused controls, recipient renal function was improved, and the length of hospital stay was shorter with a significant reduction of peak AST in the first week after transplantation (Table 1) [59,61]. While exploring the underlying molecular mechanisms, the group noted a lower expression of liver inflammatory injury indicators, including acute phase proteins, cytokines and parameters of apoptosis, adhesion molecules and oxidative stress, both at the end of preservation and after reperfusion [62]. HMP was shown to ameliorate ultrastructural changes sustained during SCS and reduced the number of CD68+ macrophages in livers [62]. The same group currently recruits participants for the PILOT study (Perfusion to Improve Liver Outcomes in Transplantation, NCT03484455) to delineate the effects of HMP in liver transplantation from retrieval to implantation using the LifePort^®^ liver transporter (Organ Recovery Systems Inc., Chicago, IL, USA) [63].

### 3.3. Hypothermic Oxygenated Perfusion (HOPE)

The HOPE technique evolved in parallel to HMP with the main difference found in the additional active perfusate oxygenation with the aim to provide oxygen to the liver at a partial pressure of 60–100 kPa [64]. The impaired mitochondrial function after warm and cold ischemia was shown to benefit from the additional active oxygenation at a level of 60–100 kPa. The positive effect on graft function and survival was also demonstrated in macrosteatotic grafts, where HOPE treatment improved mitochondrial metabolism and reduced succinate prior to normothermic reperfusion [42]. Importantly, the beneficial effect of HOPE was lost when deoxygenated perfusates were used in the same settings of macrosteatotic grafts (one-week survival of animals: 10/12 steatosis + HOPE vs. 8/12 steatosis only vs. 5/12 steatosis + non-oxygenated HMP) [42]. 

The first clinical implementation of the HOPE technique was described in 2014, when Dutkowski et al. conducted a case-matched analysis to assess the impact of HOPE treatment on Maastricht Type III-DCD allografts [65]. The group pursued this work in another analysis of 25 HOPE-perfused DCD livers which were compared to 50 unperfused cold-stored DCD livers. HOPE treatment resulted in a superior outcome regarding early liver function, peak alanine aminotransferase (ALT) levels, intrahepatic cholangiopathy, and overall biliary complications [66]. Their most recent paper analyzed the impact of HOPE perfusion on five-year outcomes in 50 patients and demonstrated similar graft survival in HOPE-perfused DCD livers and matched standard DBD grafts. Excellent five-year graft survival of 94% was reported in the HOPE-DCD group, compared to 78% in the matched SCS DCD cohort, despite a significantly higher donor risk (Table 1) [67].

Currently, five RCTs investigate this cold perfusion technique. The Zurich group initiated a multicentric RCT to assess the impact of HOPE on DBD allografts, utilizing broad inclusion criteria (e.g., retransplantations, marginal and non-marginal livers) and is powered to assess major complications (Clavien grade ≥III) (NCT01317342). The Groningen team explores the dual HOPE (d-HOPE) technique in DCD grafts. The incidence of biliary complications in DCD liver transplantation, reported as high as 25–60% [44], has prompted the development of d-HOPE for DCD grafts, aiming to achieve a better oxygen supply to the arterial peribiliary plexus. While the necessity of an additional perfusion through the hepatic artery remains a subject of ongoing debate [71], the RCT on end-ischemic d-HOPE of DCD livers has been initiated at the University of Groningen with the primary endpoint of the incidence of symptomatic NAS [72] (NCT02584283).

A multicentric RCT on HOPE application in ECD-DBD liver transplantation began recruitment in 2017 in Aachen, Germany (NCT03124641). The primary endpoint of the HOPE-ECD-DBD trial is early graft injury, assessed by peak ALT during the first seven days post-transplantation [5]. The fourth trial led by James Guarrera investigates the application of HMP from retrieval to implantation using the LifePort^®^ liver transporter (NCT03484455). A further multicenter RCT, using a two-arm design (end-ischemic HOPE vs. SCS) and aiming to recruit 266 patients receiving ECD allografts, has recently been initiated by Lesurtel et al. from the University Hospital Lyon (NCT03929523). The clinical results as well as the consecutive molecular translational analyses of these RCTs are expected to improve our understanding of IRI protection and shape the future of ECD liver transplantation (Table 2).

### 3.4. Multimodal Perfusion Approaches

Both normothermic and hypothermic perfusion approaches have demonstrated different clinical benefits. Several research groups have therefore recently postulated that the combination of cold and warm perfusion sequences, for example HOPE + NMP with gradual rewarming might further exploit the benefits of ex vivo perfusion while eliminating some inherent limitations of the different techniques [73,74]. 

Van Leeuwen et al. recently reported the clinical experience of the Groningen group with the sequential application of dual HOPE, followed by controlled oxygenated rewarming (COR), and a period of NMP [73]. Based on their previous clinical and preclinical experiences, the group hypothesized that a combination of the reconditioning effects triggered by HOPE, a subsequent transition from cold to warm perfusion using COR, and the possibility of ex situ functional assessment during NMP would further exploit the benefits of end-ischemic machine perfusion. A HOPE-COR-NMP protocol was applied to 16 discarded human livers, 11 of which met the predefined viability criteria and were successfully transplanted with 100% patient and graft survival at six months [73]. The group estimated that the introduction of d-HOPE-COR-NMP increased the number of deceased donor liver transplants by 20% in their center. To enable the transition between perfusion temperatures without the need for intermittent perfusate exchange, the authors used a hemoglobin-based oxygen carrier (Hemopure, HBOC-201, Hbo2 Therapeutics LLC, Souderton, PA, USA) [73,74]. 

### 3.5. Viability Assessment under Hypothermic Conditions and Biomarkers of IRI

Viability assessment during MP can guide the clinical decision whether to accept a liver for transplantation and is therefore an important emerging tool in ECD OLT [49]. The possibility of a reliable viability assessment is advocated as a considerable advantage of normothermic perfusion techniques [75]. By sustaining full metabolism, NMP allows to analyze several makers of liver function and injury, including biliary parameters (e.g., bile flow, bile glucose, bicarbonate, and pH), perfusate pH and base excess, portal venous and hepatic artery flow and perfusate hepatocellular enzymes [76]. Despite the reduced metabolic activity during cold storage and hypothermic liver perfusion, there is increasing evidence that a prediction of future graft performance after transplantation may be possible during HMP and HOPE [77]. Analysis of the cold perfusate during HMP/HOPE provides a unique opportunity to identity potential biomarkers which are associated with various post-OLT outcomes. A recent study involving 31 human ECD-DBD grafts initially rejected for transplantation, found that cold perfusion not only ameliorates reperfusion injury but also allows for graft viability assessment. Thus, the 2-h perfusate AST and lactate dehydrogenase (LDH) correlated significantly with the peak AST after implantation. In two grafts with a significant postreperfusion transaminase release, a high portal perfusion pressure was noted [59].

The Zurich group has recently presented a new mitochondrial marker to assess the viability of entire liver grafts during HOPE. Real-time fluorometric analysis of mitochondrial flavin mononucleotide (FMN) in the HOPE perfusate predicted human liver function, complications, and graft loss prior to transplantation [78]. The use of this surrogate parameter could facilitate proper clinical decision making on whether to accept or decline high-risk ECD allografts. This marker is currently validated in other solid organs and also in the RCT of the Guarrera group [63]. Importantly, the quantification of FMN is possible in real time, requiring only a spectroscope, and reliably predicts graft survival at a threshold of 10,000 A.U., detected within the first 30–45 min of HOPE [78]. 

In addition to the above-mentioned studies, multiple groups have extensively assessed the role of various biomarkers of inflammation, innate immunity responses, and hepatocellular injury as recently summarized in detail by Bhogal et al. [79]. 

In the near future FMN as well as other mitochondrial and inflammatory biomarkers will be assessed not only in other organs, but also during different perfusion approaches at various temperatures [78]. 

### 3.6. Allograft Therapies, Surgical Interventions, and On-Pump Drug Delivery 

The use of ex vivo machine perfusion provides a unique opportunity to selectively target the allograft and to deliver drugs and other pharmacological agents without systemic side-effects, potentially limiting complications and drug interactions in the recipient [80]. 

Several preclinical studies reported the successful delivery of various pharmacological agents, including propofol, hydrogen sulfide, carbon monoxide, nitric oxide, defatting cocktails, and nanoparticles, with the aim to modulate the release of molecules, involved in the IRI-cascade or to provide anti-oxidative protection during machine perfusion [81,82,83,84,85,86,87]. Due to the fact that most of the therapeutic interventions were tested during NMP, only limited data are available with in vivo therapeutics in HMP/HOPE [82,85,86]. Optimized perfusion solutions for HMP/HOPE containing amino acids, metabolic substrates, vitamins, and organic buffers have been investigated in kidneys and other organs showing the potential benefits in terms of post-transplant graft function [82,88,89]. 

The innate process of post-transcriptional gene regulation known as RNA interference holds promise to modulate the detrimental subcellular pathways involved in IRI. However, the systemic application of short interfering RNAs (siRNA) is associated with various challenges and high costs [86]. In a groundbreaking report, Gillooly et al. recently showed the successful transfection of siRNAs, coated in lipid nanoparticles, into hepatocytes of rat livers during hypothermic and normothermic machine perfusion [85]. This novel therapy, applied during ex vivo machine perfusion, could not only mitigate the injury transmitted through IRI, but also target other genes and pathways to, for example, reduce acute rejection and the need of systemic immunosuppression, induce tolerance, and eliminate viral solid organ contamination [86]. 

Another specific issue in liver transplantation is the need for allograft-recipient size matching and the unique ability of the liver to regenerate. In this context, machine perfusion may provide a possible platform for ex vivo splitting or size reduction of donor allografts [89,90]. 

Further research is however required to assess the role of HMP/HOPE as a potential method to deliver various targeted therapies, while bypassing the difficulty of delivering drugs, cells, and nanoparticles to the desired sites of action in systemic applications.

## 4. Molecular Effects of Hypothermic Machine Perfusion

Hypothermic machine perfusion targets different sites of the IRI cascade. While the understanding of organ protection through MP remains incomplete, several essential effects have already been identified. Hypothermic MP substantially recharges the cellular energy pool through modification of the different complexes in the respiratory chain, thereby metabolizing the accumulated succinate and other molecules [62,91].

### 4.1. The Role of the Endothelial Cells

Liver sinusoidal endothelial cells (LSECs) are regulators of liver homeostasis, and their injury is associated with impaired post-OLT liver function. In contrast to other cells and compartments, their injury is predominantly sustained during cold ischemia, where the absence of physiological blood flow-derived shear stress causes a disruption of endogenous NO production [92,93]. 

Machine perfusion confers a protective effect on LSECs, independent from the level of oxygenation [94]. Machine perfusion without additional oxygenation has improved endothelial cell integrity in a porcine DCD liver model. Although non-oxygenated HMP did not impact the high-flux mitochondrial electron transfer stages in hepatocytes, sinusoids were found with better preservation compared to static cold storage [91]. Concerns that cool perfusion temperatures reduce membrane fluidity and increase vascular resistance, thus causing unphysiological shear stress of endothelial cells, have prompted a careful adaption of perfusion pressures. Avoiding endothelial injury in the preserved liver is essential to prevent inadvertent inflammatory reactions due to adhesion molecule expression and subsequent leucocyte attachment [95]. Current clinical and experimental studies therefore control the perfusion pressure, and usually adapt it to sub-physiological levels between 20–30 mmHg in the hepatic artery, and 3–5 mmHg for the portal vein during hypothermic liver perfusion [68].

### 4.2. The Role of Cold Oxygenation

**Figure 1:**
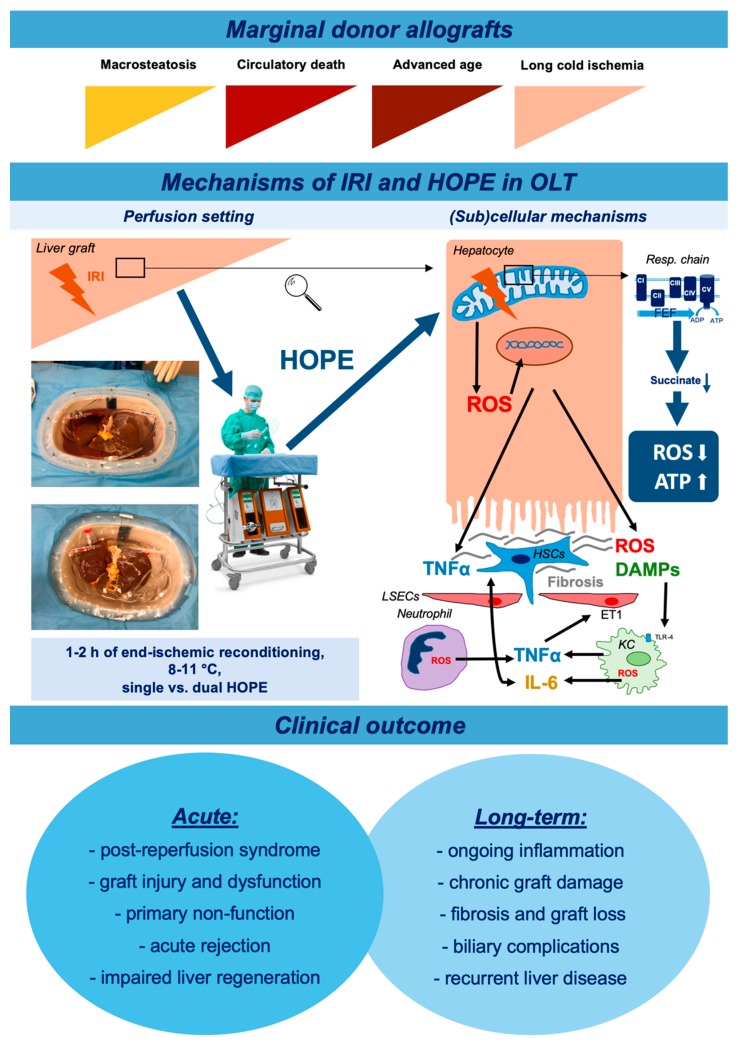
Mechanisms of ischemia-reperfusion injury and clinical application with (sub)cellular effects of hypothermic oxygenated machine perfusion. Abbreviations: IRI, ischemia-reperfusion injury; HOPE, hypothermic oxygenated machine perfusion; FEF, forward electron flow; ROS, reactive oxygen species; ADP, adenosine diphosphate; ATP, adenosine triphosphate; OLT, orthotopic liver transplantation; HSCs, hepatic stellate cells; TNFα, tumor necrosis factor alpha; LSECs, liver sinusoidal endothelial cells; DAMPs, damage associated molecular patterns; ET-1, endothelin 1; IL-6, interleukin 6; KC, Kupffer cell; TLR4, Toll-like receptor 4.

The importance of additional perfusate oxygenation was first identified in hypothermic kidney perfusion. Lazeyras et al. assessed the link between perfusate oxygenation and ATP production during cold perfusion. The authors described a required oxygen content of 100 kPa to efficiently build ATP and metabolize NADH [91,96]. Such results were paralleled by findings of the Zurich group, where hypothermically-perfused livers with deoxygenated perfusate did not recover their energy pool and experienced severe reperfusion injury following implantation [91,97]. Figure 1 highlights the underlying mechanisms of protection through cold oxygenation [77]. Reintroduction of oxygen to an ischemic graft in the cold has a distinctly different effect compared to the normothermic setting: a low rate of ROS formation coupled with the restoration of mitochondrial oxygen reserves results in markedly decreased oxidative stress at subsequent warm reperfusion [98].

Oxygen depletion during cold storage leads to a cessation of oxidative phosphorylation [99]. In a DCD pig MP model, Dutkowski et al. observed a switch from a high-flux electron transfer stage—representative of a high rate of mitochondrial respiration—to a low-flux electron transfer stage within the first 60–90 min of HOPE, as measured by the rate of NADH oxidation and CO_2_ production [91]. This effect was not observed in MP with deoxygenated perfusate, which failed to prevent mitochondrial and nuclear injury. The effect of ATP restoration during HOPE was confirmed by the Groningen group [68]. The underlying mechanism of cold oxygenation is also employed in the concept of cold oxygen persufflation, where molecular oxygen is applied in the cold [100,101].

Mitochondrial respiratory Complex I has been recognized as the major source of superoxide formation and IRI catalyzation [102]. Under physiological conditions, forward electron transport in the mitochondrial respiratory Complex I generates a protonmotive force through a redox energy difference, which eventually enables ATP synthesis at Complex V [103]. Electrons move from NADH to reduce CoQ to CoQH2, enabling protons to move across the inner mitochondrial membrane. A prerequisite for forward electron flow is a reduction potential of NAD+/NADH pool that exceeds the protonmotive force [103]. Consequently, both a diminished reduction potential and an increased protonmotive force can cause reverse electron transport. During reperfusion, several factors, such as the reduction of the CoQ pool or flavin mononucleotide (acceptor of electrons from NADH), decrease the reduction potential, while the reintroduction of oxygen triggers proton pumping into the intermembrane space by Complex III and IV and increases the protomotive force [19,104]. Oxygenation in the cold increases the reduction potential by shifting the mitochondrial redox state from reduced to oxidized [77]. Furthermore, oxygenated perfusion reduces mitochondrial electron transfer rates, thus decreasing the protonmotive force [91]. 

The group of Minor et al. compared the effect of perfusate oxygenation with air (20% oxygen) with high concentrations of perfusate oxygen (100%) in a model of 18 h cold perfusion. Importantly, the group showed a significantly reduced enzyme release during subsequent rewarming and normothermic reperfusion of livers which underwent cold perfusion with a high oxygen concentration. Additionally, lactate, a key indicator of anaerobic metabolism, was significantly lower in livers exposed to high oxygen levels in the cold. Only these livers achieved an enhanced bile production after normothermic reperfusion. Interestingly, the authors observed a free radical-mediated lipid peroxidation and activation of the AMP-activated protein kinase (AMPK) salvage pathway and upstream activation of protein kinase A after perfusion with 100% oxygen saturation [105].

Comprehensive metabolic analyses have identified the accumulation of succinate—a Complex II substrate—as the central driver of IRI [106]. Cold oxygenation has been recently shown to metabolize accumulated succinate prior to normothermic reperfusion [107]. 

Concerns that exceedingly high oxygen concentrations may confer allograft toxicity have been brought forward in the context of HOPE. These were largely based on preclinical studies, for example by Hart et al., who observed a non-significant increase in ROS formation after cold storage with 95% oxygen equilibration compared to equilibration with 21%. Both groups exhibited significantly less thiobarbituric acid reactive substances formation than the group stored in non-oxygenated solution [108]. Such results should be interpreted with caution, because the experimental settings, where an increased lipid peroxidation, reflective of ROS formation was found, involved prolonged oxygenation times (18 h vs. 24 h and 48 h) and are therefore difficult to transfer into clinical settings [105,108]. HOPE treatment is currently applied for 1–4 h in most studies and different groups explore results following long-term HOPE or d-HOPE perfusion in various countries [91].

## 5. Future Outlook and Remaining Challenges

Ischemia-reperfusion injury remains one of the leading problems in solid organ transplantation. After decades of virtually unchanged organ preservation practice, significant progress has been made in the field of ex vivo machine perfusion over the last years. Balancing wait list mortality and organ shortages, MP may ultimately lead to an expansion of the donor organ pool by incorporating ECD allografts previously deemed unsuitable for transplantation [5,109]. A central question in this context is whether an individualized approach to liver preservation will develop for the different types of ECD grafts. As such, the RCTs on HMP and HOPE already recruit distinctly different study populations [110].

Prospective clinical data comparing or combining different static and dynamic organ preservation techniques (e.g., NMP vs. HOPE and HOPE-COR-NMP) are urgently awaited to facilitate clinical application [73]. With the advent of clinical MP and the context of a dire donation situation in the Western world, it will be of utmost clinical importance to identify novel tools for allograft viability assessment and outcome prediction at various MP conditions [78]. Dynamic organ preservation techniques such as HOPE hold promise to not only enhance the performance of “marginal” ECD and DCD allografts, but also may lead to an optimized organ pool utilization. Machine perfusion techniques with further improvement of perfusates, the use of additives including pharmaceutics [111] or allograft treatment with mesenchymal stem cells during MP [87], defatting of steatotic allografts [112], and gene therapies [86], are currently being investigated to improve organ preservation and to resuscitate marginal donor allografts. 

Pushing the boundaries and exploring the potentials of MP technology—such as the seven-day perfusion of human livers with preserved function and intact liver structure [57]—raise the question of the limits of this technological development. The enthusiasm for MP has to be viewed in the context of the limitations of the current technologies, which need to be defined clearly and early enough through clinical and translational efforts. Dynamic organ preservation techniques hold promise to not only enhance the performance of “marginal” ECD and DCD allografts, but also to optimize organ pool utilization, thus improving the prognosis for patients on our waiting lists.

## Figures and Tables

**Table 1:** Published prospective clinical hypothermic machine perfusion studies in orthotopic liver transplantation (OLT) ^1^.

Author	Groups	Design	N	Donors	Perfusion Setting	Primary Endpoint	Outcome and Main Findings
Guarrera et al., 2010 [61]	HMP/SCS	case-matched	20 vs. 20	DBD, ECD grafts excluded	Modified Medtronic PBS^®^; 4–8 °C; End-ischemic HMP, dual (PV+HA)	Incidence of PNF, EAD, and patient and graft survival at 1 month and 1 year	-No significant differences in PNF, EAD, and survival but shortened hospital stay, reduced peak serum AST levels, reduced serum creatinine levels -Trends towards lower incidence of NAS
Guarrera et al., 2014 [59]	HMP/SCS	case-matched	31 vs. 30	declined ECD grafts	Modified Medtronic PBS^®^; 4–8 °C; End-ischemic HMP, dual (PV+HA)	Incidence of PNF, EAD, and vascular complication, graft and patient survival at 1 year	-Similar EAD and 1 year patient survival but shortened hospital stay, reduced peak serum AST and creatinine levels, improved early renal function -Lower incidence of biliary complications within the first year -Strong correlation with the 2 h effluent AST and LDH and peak recipient AST. High portal pressure also correlated with severity of reperfusion injury
Dutkowski et al., 2014 [65]	HOPE/SCS	case-matched	8 vs. 8	DCD (Maastricht III)	LiverAssist; 10 °C; End-ischemic HOPE, PV perfusion	Proof of clinical application of HOPE; Can HOPE rescue DCD organs?	-Clinical application of HOPE feasible and safe, even in DCD transplantation and the outcome of perfused DCD grafts is similar to matched DBD graft performance -Similar 1-year graft and patient survival -No biliary strictures in the HOPE-DCD group -Higher 6-months GFR in the HOPE group -HOPE-DCD group showed lower hospital costs
Dutkowski et al., 2015 [66]	HOPE/SCS	case-matched	25 vs. 50 DCD vs. 50 DBD	DCD (Maastricht III)	LiverAssist; 10 °C; End-ischemic HOPE, PV perfusion	Incidence and severity of biliary complications within 1 year after transplantation	-Decreased incidence of intrahepatic cholangiopathy and biliary complications in the HOPE group as well as lower rate of retransplantation for ischemic cholangiopathy and PNF -Improved 1-year graft survival in the HOPE group -Decreased peak ALT, AST, and bilirubine, less EAD, lower day 1 INR and intraoperative fresh-frozen plasma transfusions -Lower 3 and 6 months ALP and 6-months bilirubin -Trends in better renal function, ICU and hospital stay, HAT, acute rejection, and PNF -HOPE DCD livers achieved similar results as matched DBD livers in all investigated endpoints
van Rijn et al., 2017 [68]	DHOPE/SCS	prospective case-control study	10 vs. 32	DCD (Maastricht III)	LiverAssist; 10 °C; End-ischemic HOPE, dual (PV + HA)	Graft survival at 6 months after OLT (time from transplantation to retransplantation or death from graft failure)	-Higher 6 months graft survival in HOPE group vs. SCS -Safety and feasibility of dual HOPE -Increase of hepatic ATP content during HOPE and lower peak serum ALT and lower day 7 post-OLT bilirubin -Lower median ALT, gamma GT, ALP, and bilirubin serum levels 30 days after OLT -Trends to a lower incidence of NAS and in length of ICU or hospital stay -Higher incidence of hypokalemia after reperfusion in HOPE group
van Rijn et al., 2018 [69]	d-HOPE/SCS	prospective phase I stury	10 vs. 20	DCD	LiverAssist; 10 °C; End-ischemic HOPE, dual (PV + HA)	Histological biliary injury based on bile duct biopsies	-The reduced bile-duct injury and less injury of the deep peribiliary glands in d-HOPE-preserved livers
Schlegel et al., 2019 [67]	HOPE/SCS	case-matched	50 vs. 50 DCD vs. 50 DBD	DCD (Maastricht III)	LiverAssist; 10–12 °C; End-ischemic HOPE, dual (PV + HA)	Post-transplant complications, and non-tumor-related patient death or graft loss	-Similar graft survival in HOPE-DCD livers like in DBD -Five-year graft survival was 94% after HOPE-DCD vs. 78% in untreated DCD
Patrono et al., 2019 [70]	d-HOPE/SCS	case-matched	25 vs. 50	DBD	LiverAssist; 10 °C; End-ischemic HOPE, dual (PV + HA)	Multiple clinical endpoints	-HOPE was associated with a lower severe post-reperfusion syndrome rate and stage 2–3 acute kidney injury -Lower transaminases peak and a lower early allograft dysfunction (EAD) rate after HOPE -A steeper decline in arterial graft resistance throughout perfusion was associated with lower EAD rate
van Leeuwen et al., 2019 [60]	d-HOPE-COR-NMP	prospective single arm	16	DCD	LiverAssist; 8–12 °C End-ischemic HOPE, dual (PV + HA), followed by COR and NMP	3-months graft survival	-All livers (*n* = 11) which met viability criteria were transplanted successfully with 100% 6-months survival -Introduction of HOPE-COR-NMP increased the number of transplantations by 20%

^1^ Only completed clinical trials are shown. Case reports and unstructured single center experiences are excluded from the table. Only main aspects of the study are detailed. Listed perfusion devices: Medtronic PBS (Minneapolis, MI, USA); LiverAssist, Organ Assist (Groningen, The Netherlands); Exiper, Bologna Machine Perfusion (Medica s.p.a., Bologna, Italy). Abbreviations used: ALP, alkaline phosphatase; AST, aspartate aminotransferase; ALT, alanine aminotransferase; COR, controlled oxygenated rewarming; DBD, donation after brain death; DCD, donation after circulatory death; d-HOPE, dual hypothermic oxygenated machine perfusion; EAD, early allograft dysfunction; ECD, extended criteria donation; HA, hepatic artery; HAT, hepatic artery thrombosis; HMP, hypothermic machine perfusion; HOPE, hypothermic oxygenated machine perfusion; ICU, intensive care unit; INR, international normalized ratio; IRI, ischemia-reperfusion injury; kPa, kilopascal; LDH, lactate dehydrogenase; MP, machine perfusion; NAS, non-anastomotic biliary strictures; NMP, normothermic machine perfusion; OLT, orthotopic liver transplantation; PNF, primary non-function; PV, portal vein; SCS, static cold storage.

**Table 2:** Overview of currently ongoing randomized control trials (RCTs) on hypothermic machine perfusion (MP) in liver transplantation.

Group/NCT	MP and Comp.	Design	N	Donors	Perfusion ^1^	Primary Endpoint
Zurich, Switzerland NCT01317342	HOPE/SCS	recruitment completed multicenter RCT	85 vs. 85	DBD	LiverAssist; 8–10 °C; end-ischemic HOPE, single (PV) perfusion	Major postoperative complications (Clavien grade ≥III) and CCI
Groningen, Netherlands NCT02584283	DHOPE/SCS	recruitment completed multicenter RCT	78 vs. 78	DCD (Maastricht category III)	LiverAssist; 8–10 °C; end-ischemic dual (PV+HA) HOPE, 2 h	Incidence of NAS
Aachen, Germany NCT03124641	HOPE/SCS	Recruiting multicenter RCT	23 vs. 23	ECD-DBD	LiverAssist; 8–10 °C; end-ischemic HOPE, single (PV) perfusion, 1(–2) h	Early graft injury (peak ALT level)
New Jersey, USA NCT03484455	HMP/SCS	Recruiting multicenter RCT	70 vs. 70	Not stated	LifePort Liver Transporter, Temperature not stated; preservation HMP, dual perfusion from retrieval to implantation, no active oxygenation, 3–7 h	Early allograft dysfunction (EAD)
Lyon, France NCT03929523	HMP/SCS	Recruiting multicenter RCT	133 vs. 133	ECD-DBD	LiverAssist; 8–10 °C; end-ischemic HOPE, single (PV) perfusion, 1(–2) h	Early allograft dysfunction (EAD)

^1^ Listed perfusion devices: LiverAssist, Organ Assist (Groningen, The Netherlands); LifePort Liver Transporter, Organ Recovery Systems (Chicago, IL, USA). Abbreviations: RCT, randomized controlled trial; CCI, comprehensive complication index; ALT, alanine aminotransferase; DBD, donation after brain death; DCD, donation after circulatory death; d-HOPE, dual hypothermic oxygenated machine perfusion; EAD, early allograft dysfunction; ECD, extended criteria donation; HA, hepatic artery; HOPE, hypothermic oxygenated machine perfusion; MP, machine perfusion; NAS, non-anastomotic biliary strictures; PV, portal vein; SCS, static cold storage.
